# Polymorphisms in the *CCR5* promoter associated with cervical intraepithelial neoplasia in a Chinese Han population

**DOI:** 10.1186/s12885-019-5738-6

**Published:** 2019-05-31

**Authors:** Shuyuan Liu, Jun Chen, Zhiling Yan, Shuying Dai, Chuanyin Li, Yufeng Yao, Li Shi

**Affiliations:** 1 Institute of Medical Biology, Chinese Academy of Medical Sciences & Peking Union Medical College, Yunnan Key Laboratory of Vaccine Research & Development on Severe Infectious Disease, Kunming, 650118 Yunnan China; 2 The Third People’s Hospital of Kunming, Kunming, 650041 China; 3 School of Basic Medical Science, Kunming Medical University, Kunming, 650500 China; 4 School of Pharmaceutical Science, Yunnan Key Laboratory of Pharmacology for Natural Products, Kunming Medical University, Kunming, 650500 China

**Keywords:** Cervical carcinogenesis, Cervical intraepithelial neoplasia, *CCR5* gene, Promoter polymorphism

## Abstract

**Background:**

C-C chemokine receptor 5 (CCR5) has attracted wide concern for its critical role in the progression of human immunodeficiency virus type 1 (HIV-1) infection. Several studies have demonstrated that CCR5 affects the processes of tumor cell migration, invasion, and metastasis. The aim of this study was to illustrate the association between the polymorphisms of the CCR5 promoter and the development of cervical cancer.

**Methods:**

336 women with cervical intraepithelial neoplasia (CIN), 488 women with cervical cancer (CC), and 682 healthy controls were recruited to detect polymorphisms in the *CCR5* promoter using a sequencing method.

**Results:**

Six loci with polymorphism were found in the *CCR5* promoter; the frequencies of the minor alleles of rs1799987 was significantly higher in the CIN group than that in the control group (*P* = 0.007); and the genotypic frequencies of rs2734648, rs1799987, rs1799988 and rs1800023 were significantly different between the CIN group and the control group (*P* < 0.008). The inheritance model analysis showed that rs2734648, rs1799987, rs1799988 and rs1800023 significantly increased the susceptibility to CIN in a recessive genetic model (*P* < 0.008). The haplotype constructed by the major alleles of these 6 SNPs (rs2227010-rs1799987-rs1799988-rs2734648-rs1800023-rs1800024: A-G-A-C-A-T) was highly protective against CIN (OR = 0.731, 95%CI: 0.603–0.886, *P* = 5.68E-03). In addition, transcription prediction showed that mutation of these 6 SNPs might alternate the binding of particular transcription factors.

**Conclusion:**

The *CCR5* promoter polymorphisms were significantly associated with cervical intraepithelial neoplasia by altering the expression of *CCR5* on the cell surface in a Chinese Han population.

## Background

C-C motif chemokine receptor type 5 (CCR5), a transmembrane G-coupled cell-surface chemokine receptor, binds to five kinds of CC-chemokines: human macrophage inflammatory protein-1α (MIP-1α), MIP-1β, RANTES (regulated on activation, normal T cell expressed and secreted), monocyte chemotactic protein 2 (MCP-2) and MCP-4 [1, 2]. CCR5 is known to be the principal coreceptor of macrophage-tropic (R5) strains of human immunodeficiency virus-type 1 (HIV-1), which is highly variably expressed on the cell surface of the memory T cells, macrophages, dendritic cells, hematopoietic stem cells, and microglial cells [1, 3].

The human *CCR5* gene located on 3p21.31, is composed of four exons and two introns. The promoter region has been described previously [4, 5], and the differences in the promoter region may regulate the expression of CCR5 in monocyte/macrophages and T lymphocytes [5]. *CCR5* expression in tumor cells and various host cells plays a very important role in tumor progression [6]. Studies have demonstrated that CCR5 and CCR5 ligands CCL5 might stimulate angiogenesis as growth factors, modulate the recruitment of inflammatory cells, and induce the tumor immune evasion [6, 7]. It has been reported that men with a loss of functional CCR5 and carrying the *CCR5* Δ32 mutant are resistant to the development of prostate cancer. Additionally, the expression of the CCR5 ligand CCL5 promotes the migration and invasiveness of pancreatic cancer [8].

Cervical carcinoma is the second most common cancer affecting women worldwide [9]. Approximately 75,000 women develop cervical cancer, and 40,000 women die from this disease in China each year [10]. The development of cervical carcinoma is strongly linked to persistent infection of high-risk human papillomaviruses (HPV), such as HPV16 and HPV18 [11]. Most HPV infections do not cause any symptoms or disease and clear up spontaneously; however, a minority of women exhibit persistent HPV infection, which might lead to cervical intraepithelial neoplasia (CIN) or cervical cancer (CC) [12, 13], which means that the host immune system and genetic background play important roles in the progression of cervical cancer [14]. In recent years, Che et al [15] and Sales et al [16] found that *CCR5* expression is extremely higher in cervical cancer tissue than in matched normal control tissue and that downregulation of *CCR5* suppresses cervical cancer cell growth and proliferation in vivo [15]. Variability of the promoter region of *CCR5* gene might be the reason for differing *CCR5* expression levels.

In the present study, we detected the genetic polymorphisms in the promoter region of *CCR5* of a Chinese Han population and investigated the association between genetic polymorphisms of *CCR5* gene promoter and cervical cancer in Han Chinese.

## Methods

### Subjects

A total of 336 cervical intraepithelial neoplasia (CIN) and 488 cervical cancer (CC) female patients were enrolled in the Third Affiliated Hospital of Kunming Medical University (Kunming, China) from 2011 to 2015. All subjects were Han Chinese in Yunnan province (Southwest China).

Diagnoses of CIN and CC were based on comprehensive cervical cancer control guidelines from the World Health Organization (WHO) [17]. Patients undergoing any anti-cancer therapy, such as radiotherapy and chemotherapy before surgery, suffering from other malignant tumors, or with cardiovascular disease, diabetes, hepatitis, kidney disease, etc. were excluded.

In the same period, 682 healthy women were recruited as a control group. The control subjects were healthy women without any history of abnormal Pap Smear or cervical lesions and other cancers on the day of recruitment.

### DNA extraction and sequencing

The genomic DNA of all patients was extracted from the peripheral lymphocytes using the QIAamp Blood Mini Kit (Qiagen, Hilden, Germany).

**Fig. 1:**
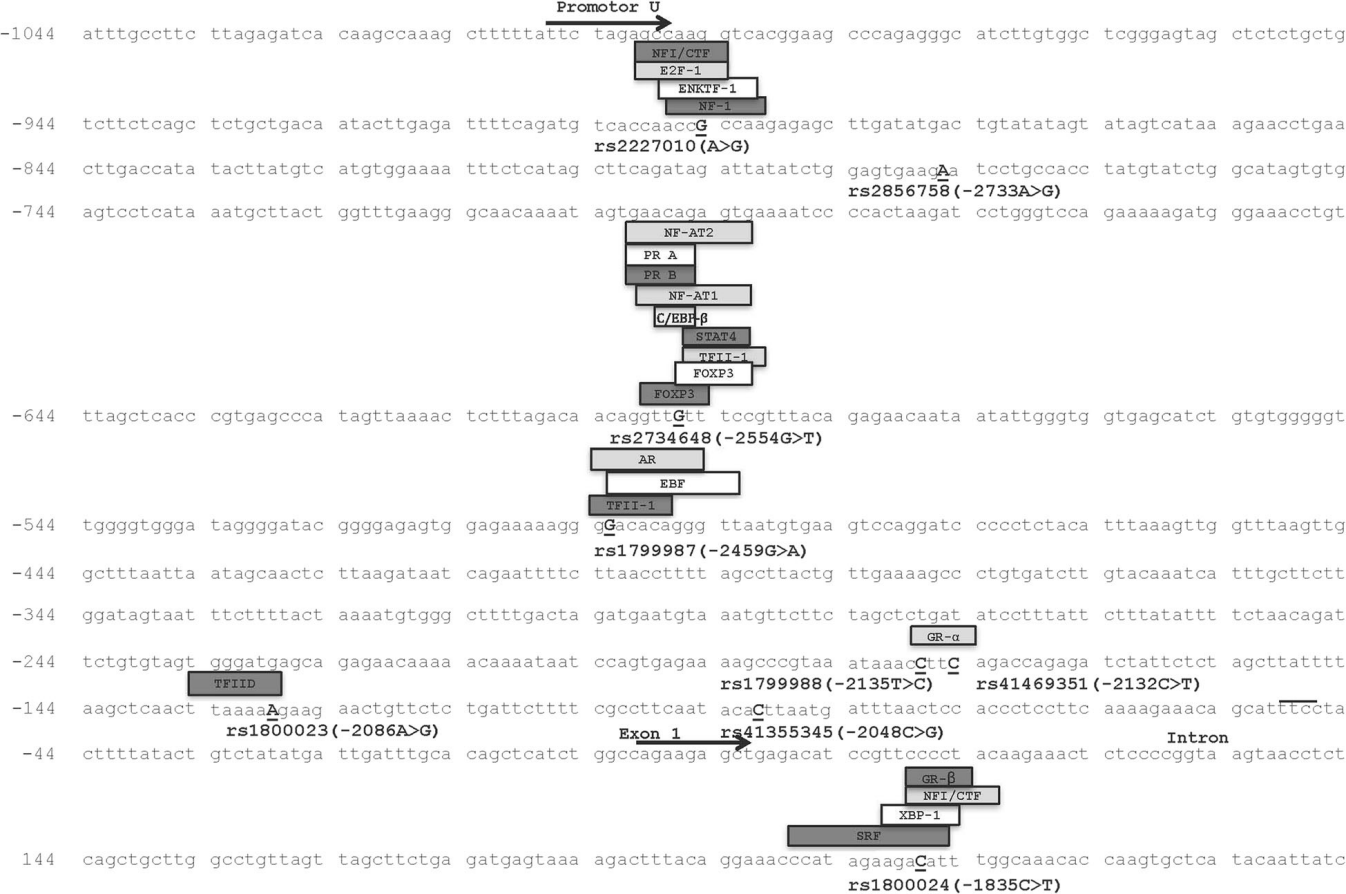
Sequence of *CCR5* promoter. Single nucleotide polymorphisms (SNPs) were marked in the sequence and predicted transcriptional factors were also marked in the sequence. Abbreviations: NF, nuclear factor; CTF, site-specific DNA-binding proteins or CAAT box transcription factor; E2F-1, adenovirus E2 gene promoter region binding factor 1; ENKTF-1, enkephalin transcription factor-1; PR, progesterone receptor; C/EBP, CCAAT/enhancer-binding protein; STAT4, signal transducers and activators of transcription; TF, transcription factor; FOXP3, forkhead box P3; AR, androgen receptor; EBF, early B-cell factor; GR-α, glucocorticoid receptor; CTF, CCAAT box-binding transcription factor; XBP-1, x box binding protein 1; SRF, seurm response factor

The CCR5 promoter region was PCR-amplified using the following primers, CCR5P_F: 5′-gacgagaaagctgagggtaaga-3′ and CCR5P_R: 5′-taaccgtctgaaactcattcca-3′, and PCR fragment was 1388 bp (Fig. 1). PCR for each sample was carried out in a 50-μL reaction volume containing 10 ng of DNA, 10 pmol of each primer, 1.25 U ExTaq polymerase (TaKaRa, Dalian, China), 200 mmol/L dNTPs (TaKaRa) and 1 × ExTaq PCR buffer (TaKaRa). Amplification consisted of an initial denaturation step of 5 min at 98 °C; 30 cycles of 10 s of denaturation at 98 °C, 5 s of annealing at 55 °C, extension at 72 °C for 90 s; and a final extension for 5 min at 72 °C. The purified PCR fragment was sequenced to detect the polymorphism loci in this region, using the same primers as in PCR reaction, with Big Dye Terminator Reaction Mix (Applied Biosystems, Foster City, CA, USA). The reaction products were purified by Big Dye XTerminator Purification Kit (Applied Biosystems) and run on ABI 3730XL sequencer. Sequencing results were analyzed using the DNASTAR Lasergene v7.1 package.

### Transcription prediction

PROMO version 8.3 (http://alggen.lsi.upc.es/cgi-bin/promo_v3/promo/promoinit.cgi?dirDB=TF_8.3) was used to predict the potential binding sites in *CCR5* promoter of transcription factors [18, 19].

### Statistical analysis

The ages among different groups were compared using one-way ANOVA, followed by an LSD test for multiple comparisons correction. Basic statistical analysis for allele and genotype and disease association was performed using PLINK v1.9 (http://zzz.bwh.harvard.edu/plink/data.shtml) [20].

All polymorphic loci were tested for deviation from the Hardy-Weinberg equilibrium in the control group with a threshold of 0.05. Association tests for allelic and genotypic frequencies, as well as dominant and recessive inheritance models of these SNPs among CIN, CC and control groups using the χ^2^-test were also performed by PLINK. Risks were estimated by the odds ratios (OR) with 95% confidence interval (95%CI). Linkage disequilibrium (LD) among these SNPs was also estimated, where the linkage disequilibrium coefficient D was calculated using Haploview 4.2 software. LD is displayed as pairwise D’, and the D’ values defined in the range [− 1, 1], with a value of 1 representing perfect disequilibrium. The D’ value over 0.8 indicated the existence of different loci in the LD. The differences in the haplotypes between the case and control groups were determined with the χ^2^-test. Power-analysis was performed using Power and Sample Size Calculations (version 3.1.2) [21]. Significance threshold after Bonferroni correction for multiple comparisons is *P* value less than 0.008.

## Results

### Characteristics of subjects

There were 336 CIN patients and 488 CC patients compared with 682 normal women. The mean age was 48.122 ± 9.578 (ranged from 22 to 75) years in CIN group, 48.180 ± 9.655 (ranged from 24 to 73) years in the CC group, and 48.934 ± 7.390 (ranged from 33 to 79) years in the control group. No significant differences in age were found in the pairwise comparisons of CIN, CC, and control group (*P* > 0.05).

### Polymorphism loci in CCR5 promoter region

According to GenBank (NC_000003.12), there are 9 identical single-nucleotide polymorphism (SNP) loci in the promoter of *CCR5* (Fig. 1): rs2227010 (A > G), rs2856758 (A > G), rs2734648 (T > G), rs1799987 (G > A), rs1799988 (T > C), rs41469351 (C > T), rs1800023 (G > A), rs41355345 (C > G) and rs1800024 (C > T). Six SNPs (rs2227010, rs2734648, rs1799987, rs1799988, rs1800023 and rs180024) were found with polymorphism in the Chinese Han population, whereas all participants were AA at rs2856758, CC at rs41469351 and CC at rs41355345.

### Association of polymorphism in CCR5 promoter region with cervical cancer

All these 6 SNPs with polymorphism were in the Hardy-Weinberg equilibrium (HWE) in the control group (*P* > 0.05). The distribution of allelic and genotypic frequencies of these 6 SNPs among CIN, CC and control group are presented in Tables 1 and 2, the frequencies of the G-allele of rs2734648, were significantly higher in the CIN group than in the control group (*P* = 0.007, OR = 1.289, 95%CI: 1.071–1.551, Table 1). The genotypic frequencies of rs2734648, rs1799987, rs1799988 and rs1800023 were distinctly different between the CIN group and the control group (*P* < 0.008, Table 2). In addition, no significant differences in allelic or genotypic frequencies were found between the CC and control groups.

**Table 1:** Allelic distribution of SNPs in CCR5 promoter among control, CIN and CC groups

		CO (*N* = 682)	CIN (*n* = 336)	CC (*n* = 488)	CIN vs CO		CC vs CO		CIN vs CC	
					P*	OR(95%CI)	*P*	OR(95%CI)	*P*	OR(95%CI)
rs2227010	G	244 (17.89%)	142 (21.13%)	185 (18.95%)	0.079	1.230 (0.976–1.550)	0.511	1.074 (0.869–1.327)	0.276	0.873 (0.683–1.115)
	A	1120 (82.11%)	530 (78.87%)	791 (81.05%)						
rs2734648	G	622 (45.60%)	349 (51.93%)	480 (49.18%)	**0.007**	1.289 (1.071–1.551)	0.087	1.154 (0.979–1.361)	0.272	1.117 (0.917–1.359)
	T	742 (54.40%)	323 (48.07%)	496 (50.82%)						
rs1799987	A	557 (40.84%)	312 (46.43%)	428 (43.85%)	0.016	1.256 (1.042–1.513)	0.145	1.132 (0.958–1.336)	0.302	0.901 (0.740–1.098)
	G	807 (59.16%)	360 (53.57%)	548 (56.15%)						
rs1799988	C	579 (42.44%)	315 (46.88%)	415 (42.52%)	0.058	1.196 (0.994–1.440)	0.972	1.003 (0.849–1.184)	0.08	0.838 (0.688–1.022)
	T	785 (57.55%)	357 (53.12%)	561 (57.48%)						
rs1800023	A	622 (45.60%)	345 (51.34%)	472 (48.36%)	0.015	1.259 (1.046–1.514)	0.187	1.117 (0.948–1.317)	0.235	0.888 (0.729–1.080)
	G	742 (54.40%)	327 (48.66%)	504 (51.64%)						
rs1800024	T	320 (23.46%)	173 (25.74%)	235 (24.08%)	0.258	1.131 (0.914–1.400)	0.729	1.035 (0.853–1.255)	0.441	0.915 (0.729–1.148)
	C	1044 (76.54%)	499 (74.26%)	741 (75.92%)						

*Abbreviations*: *SNP* Single nucleotide polymorphism, *CIN* Cervical intraepithelial neoplasia, *CC* Cervical cancer, *CO* Control, *vs* Versus, *OR* Odds ratio, *CI* Confidence Interval

*Significance threshold after Bonferroni correction for multiple comparisons is *P* < 0.008 (which was indicated in bold)

**Table 2:** Genotypic distribution of SNPs in CCR5 promoter among control, CIN and CC groups

		Control (N = 682)	CIN (n = 336)	CC (n = 488)	CIN vs CO	CC vs CO	CIN vs CC
rs2227010	GG	28 (4.11%)	11 (3.27%)	24 (4.92%)	χ^2^ = 7.174, P* = 0.028	χ^2^ = 0.522, *P* = 0.770	χ^2^ = 6.099, *P* = 0.047
	GA	188 (27.56%)	120 (35.72%)	137 (28.07%)			
	AA	466 (68.33%)	205 (61.01%)	327 (67.01%)			
rs2734648	GG	143 (20.97%)	109 (32.44%)	131 (26.84%)	χ^2^ = 17.260, ***P*** **= 1.78E-04**	χ^2^ = 5.623, *P* = 0.060	χ^2^ = 3.658, *P* = 0.161
	TG	336 (49.26%)	131 (38.99%)	218 (44.67%)			
	TT	203 (29.77%)	96 (28.57%)	139 (28.48%)			
rs1799987	AA	121 (17.74%)	93 (27.68%)	110 (22.54%)	χ^2^ = 14.59, ***P*** **= 6.78E-04**	χ^2^ = 4.249, *P* = 0.1195	χ^2^ = 3.420, *P* = 0.181
	GA	315 (46.19%)	126 (37.50%)	208 (42.62%)			
	GG	246 (36.07%)	117 (34.82%)	170 (34.84%)			
rs1799988	CC	129 (18.91%)	91 (27.08%)	96 (19.67%)	χ^2^ = 9.808, ***P*** **= 0.007**	χ^2^ = 0.231, *P* = 0.891	χ^2^ = 6.636, *P* = 0.036
	TC	321 (47.07%)	133 (39.59%)	223 (45.70%)			
	TT	232 (34.02%)	112 (33.33%)	169 (34.63%)			
rs1800023	AA	147 (21.55%)	105 (31.26%)	127 (26.02%)	χ^2^ = 11.89, ***P*** **= 0.003**	χ^2^ = 3.246, *P* = 0.1973	χ^2^ = 2.904, *P* = 0.234
	GA	328 (48.10%)	135 (40.17%)	218 (44.67%)			
	GG	207 (30.35%)	96 (28.57%)	143 (29.30%)			
rs1800024	TT	43 (6.30%)	24 (7.14%)	26 (5.33%)	χ2 = 1.312, *P* = 0.519	χ2 = 1.510, *P* = 0.470	χ2 = 1.166, *P* = 0.558
	CT	234 (34.32%)	125 (37.21%)	183 (37.50%)			
	CC	405 (59.38%)	187 (55.65%)	279 (57.17%)			

*Abbreviations*: *SNP* Single nucleotide polymorphism, *CIN* Cervical intraepithelial neoplasia, *CC* Cervical cancer, *CO* Control, *vs* Versus

*Significance threshold after Bonferroni correction for multiple comparisons is *P* < 0.008 (which was indicated in bold)

Our study had a power over 80% to detect and odds ratios (ORs) of 1.403 for rs2227010, 1.971 for rs2734648, 1.963 for rs1799987, 1.898 for rs1799988, 1.906 for rs1800023, and 1.661 for rs1800024, considering the sample size of cases and controls and assuming the minor allele frequencies of each SNP in the Han Chinese population of Yunnan.

### Inheritance model analysis among the CIN, CC and control groups

We also applied dominant and recessive inheritance model analysis to investigate the association between these 6 SNPs in the *CCR5* promoter and cervical cancer development. The results showed that rs2734648, rs1799987, rs1799988 and rs1800023 significantly increased the susceptibility to CIN in the recessive inheritance model (*P* < 0.008, Table 3); and rs2734648-GG, rs1799987-AA, rs1799988-CC and rs1800023-AA were the risk genotypes to CIN.

**Table 3:** Different inheritance model analysis among CIN, CC and control groups

Inheritance Model		CO (n = 682)	CIN (n = 336)	CC (n = 488)	CIN vs CO		CC vs CO		CIN vs CC	
					OR (95% CI)	P*	OR (95% CI)	*P*	OR (95% CI)	*P*
rs2227010										
Dominant	A/A	466 (68.33%)	205 (61.01%)	327 (67.01%)	1.379 (1.050–1.810)	0.021	1.062 (0.829–1.362)	0.634	0.770 (0.577–1.029)	0.077
	A/G-G/G	216 (31.67%)	131 (38.99%)	161 (32.99%)						
Recessive	A/A-A/G	654 (95.89%)	325 (96.73%)	464 (95.18%)	0.791 (0.389–1.608)	0.516	1.208 (0.691–2.111)	0.506	1.528 (0.738–3.164)	0.25
	G/G	28 (4.11%)	11 (3.27%)	24 (4.92%)						
rs2734648										
Dominant	T/T	203 (29.77%)	96 (28.57%)	139 (28.48%)	1.059 (0.794–1.413)	0.694	1.064 (0.824–1.375)	0.635	1.004 (0.738–1.366)	0.978
	G/T-G/G	479 (70.23%)	240 (71.43%)	349 (71.52%)						
Recessive	T/T-G/T	539 (79.03%)	227 (67.56%)	357 (73.16%)	1.810 (1.350–2.427)	**6.66E-05**	1.383 (1.054–1.816)	0.019	0.764 (0.564–1.035)	0.082
	G/G	143 (20.97%)	109 (32.44%)	131 (26.84%)						
rs1799987										
Dominant	G/G	246 (36.07%)	117 (34.82%)	170 (34.84%)	1.056 (0.803–1.388)	0.696	1.055 (0.828–1.346)	0.664	0.999 (0.747–1.338)	0.997
	A/G-A/A	436 (63.93%)	219 (65.18%)	318 (65.16%)						
Recessive	G/G-A/G	561 (82.26%)	243 (72.32%)	378 (77.46%)	1.774 (1.302–2.418)	**2.5E-04**	1.349 (1.010–1.802)	0.042	0.760 (0.552–1.047)	0.093
	A/A	121 (17.74%)	93 (27.68%)	110 (22.54%)						
rs1799988										
Dominant	T/T	232 (34.02%)	112 (33.33%)	169 (34.63%)	1.031 (0.782–1.360)	0.828	1	0.827	0.944 (0.704–1.266)	0.699
	C/T-C/C	450 (65.98%)	224 (66.67%)	319 (65.37%)			0.973 (0.762–1.243)			
Recessive	T/T-C/T	553 (81.09%)	245 (72.92%)	392 (80.33%)	1.592 (1.171–2.166)	**0.003**	1	0.746	0.659 (0.475–0.915)	0.013
	C/C	129 (18.91%)	91 (27.08%)	96 (19.67%)			1.050 (0.782–1.409)			
rs1800023										
Dominant	G/G	207 (30.35%)	96 (28.57%)	143 (29.30%)	1.089 (0.817–1.452)	0.559	1	0.699	0.965 (0.710–1.311)	0.82
	A/G-A/A	475 (69.65%)	240 (71.43%)	345 (70.70%)			1.051 (0.815–1.356)			
Recessive	G/G-A/G	535 (78.45%)	231 (68.75%)	361 (73.98%)	1.654 (1.233–2.220)	**7.5E-04**	1	0.075	0.774 (0.569–1.052)	0.101
	A/A	147 (21.55%)	105 (31.25%)	127 (26.02%)			1.280 (0.975–1.681)			
rs1800024										
Dominant	C/C	405 (59.38%)	187 (55.65%)	279 (57.17%)	1.165 (0.895–1.517)	0.257	1	0.449	0.940 (0.710–1.244)	0.666
	C/T-T/T	277 (40.62%)	149 (44.35%)	209 (42.83%)			1.095 (0.865–1.386)			
Recessive	C/C-C/T	639 (93.70%)	312 (92.86%)	462 (94.67%)	1.143 (0.681–1.918)	0.612	1	0.484	0.732 (0.412–1.298)	0.284
	T/T	43 (6.30%)	24 (7.14%)	26 (5.33%)			0.836 (0.507–1.381)			

*Abbreviations*: *CIN* Cervical intraepithelial neoplasia, *CC* Cervical cancer, *CO* Control, *vs* Versus, *OR* Odds ratio, *CI* Confidence Interval

*Significance threshold after Bonferroni correction for multiple comparisons is *P* < 0.008 (which was indicated in bold)

### Haplotype analysis among CIN, CC and control group

The LD among these 6 SNPs was estimated, where the linkage disequilibrium coefficient D (D’) was calculated, and the D’ value of these 6 SNPs was over 0.8, which indicated that these 6 SNPs of CCR5 promoter region were in LD.

Then, we constructed the haplotype of these 6 SNPs (rs2227010-rs1799987-rs1799988-rs2734648-rs1800023-rs1800024) and pairwise compared the haplotype frequencies among CIN, CC and control groups. Table 4 presents 4 different haplotypes (H1, H2, H3 and H4) with frequencies over 3%. Haplotype H1 (A-T-G-T-G-C) was the most common haplotype in CIN patients (45.39%), CC patients (47.54%) and controls (50.58%), and the frequency of H1 was significantly lower in the CIN group compared with the control group (OR = 0.731, 95%CI: 0.603–0.886, *P* = 1.42E-03). All 4 kinds of haplotypes showed no differences between the CC and control groups, or between the CIN and CC groups.

**Table 4:** haplotype frequencies comparison among CIN, CC and control groups

Haplotypes^a^	CO (freq.)	CIN (freq.)	CC (freq.)	CIN vs CO		CC vs CO		CIN vs CC	
				P*	OR (95%CI)	*P*	OR (95%CI)	*P*	OR (95%CI)
H1: A T G T G C	690 (50.58%)	305 (45.39%)	464 (47.54%)	1.42E-03	**0.731 (0.603~0.886)**	0.060	0.847 (0.713~1.007)	0.157	1.159 (0.945~1.421)
H2: A G A C A T	282 (20.67%)	161 (23.95%)	217 (22.23%)	0.21	1.153 (0.923~1.441)	0.486	1.075 (0.877~1.316)	0.558	0.932 (0.737~1.179)
H3: G G A C A C	214 (15.69%)	134 (19.94%)	162 (16.60%)	0.042	1.284 (1.009~1.634)	0.635	1.056 (0.843~1.323)	0.133	0.822 (0.637~1.062)
H4: A G G T A C	44 (3.23%)	31 (4.61%)	50 (5.12%)	0.178	1.380 (0.862~2.210)	0.026	1.598 (1.056~2.418)	0.533	1.158 (0.730~1.836)

*Abbreviations*: *CIN* Cervical intraepithelial neoplasia, *CC* Cervical cancer, *CO* Control, *vs* Versus, *OR* Odds ratio, *CI* Confidence Interval, *H* Haplotype

^a^haplotype, constructed by rs2227010-rs1799987-rs1799988-rs2734648-rs1800023-rs1800024

*Significance threshold after Bonferroni correction for multiple comparisons is *P* < 0.008 (which was indicated in bold)

### Transcription prediction

PROMO version 8.3 (http://alggen.lsi.upc.es/cgi-bin/promo_v3/promo/promoinit.cgi?dirDB=TF_8.3) was used to predict the potential binding sites of transcription factors in the CCR5 promoter region [18, 19], the computer algorithms suggested that these 6 SNPs might be binding sites for particular transcription factors (Fig. 1).

## Discussion

Tumor development and progression depend heavily on the presence of angiogenesis factors including chemokines and their receptors [22]. There has been evidence that chemokines and their receptors expressed on tumor cells contribute to recruitment and activation of tumor-suppressor cells [6, 23] and recruitment of inflammatory cells to the tumor microenvironment [24], which may induce the process of tumor cell migration, invasion, and metastasis [22, 24–26]. As one of the key chemokines involved in many pathological processes, including inflammation, angiogenesis, tumor cell growth and infiltration, *CCR5* may influence the cervical cancer progression. In the current study, we performed the association study of *CCR5* promoter (P_1_) polymorphisms with cervical carcinoma and found that the promoter variations of *CCR5* might be associated with development of cervical lesions, cervical intraepithelial neoplasia.

Two *CCR5* promoters have been previously described: a weak upstream promoter called P_U_ or P_2_, and a stronger downstream promoter called P_D_ or P_1_ [4, 27]. The expression of *CCR5* on cell surface is highly variable, and *CCR5* promoter polymorphisms may alter the *CCR5* cell surface expression, which might consequently influence the disease progression. Previously, several SNPs in the *CCR5* promoter have been shown to affect *CCR5* expression. Salkowitz et al found that rs1799987 (−2459G > A) located in the P_1_ was related to a different expression level of CCR5 on CD14^+^ monocytes, and individuals carrying homozygous rs1799987-G exhibited lower CCR5 density on CD14^+^ monocytes [28]. Mummidi et al [29] reported that rs1799987 was associated with the rate of AIDS progression. *CCR5* promoter polymorphisms correlate with HIV disease progression possibly by regulating CCR5 cell surface expression and CD4^+^ T cell apoptosis in HIV patients. In this study, rs1799987-AA significantly increased the risk of susceptibility to CIN, which indicated that rs1799987-AA might be a risk factor for cervical lesions development, possibly by influencing the expression level of CCR5.

*CCR5* promoter haplogroups constructed by -2733A/G, −2554G/T, −2459G/A, − 2135 T/C, −2132C/T, −2086A/G, −1835C/T and + 554(Δ32), have been characterized and described before, and CCR5 haplogroups have been named as CCR5-HHA (A-G-G-T-C-A-C-Δ32wild), −HHB (A-T-G-T-C-A-C-Δ32wild), −HHC (A-T-G-T-C-G-C-Δ32wild), −HHD (A-T-G-T-T-A-C-Δ32wild), −HHE (A-G-A-C-C-A-C-Δ32wild), −HHF (A-G-A-C-C-A-T-Δ32wild), −HHG*1 (G-G-A-C-C-A-C-Δ32wild) and -HHG*2 (G-G-A-C-C-A-C-Δ32mutant) [27, 30, 31]. A specific *CCR5* promoter haplogroup was demonstrated to correlate with transcriptional activity [27] and affect the rate of AIDS progression [30]. CCR5-HHA haplotype is associated with lower CCR5 expression and protective phenotype in humans, whereas CCR5-HHF or CCR5-HHG is related to higher CCR5 expression [32]. We performed haplotype analysis with 6 polymorphism loci in *CCR5* promoter, and only SNP rs2227010 was not comprised in the defined *CCR5* haplogroups. Our results showed that haplotype H1: A-T-G-T-G-C, which was similar to CCR5-HHC, was the most-common haplotype in our participants and consisted of the major alleles of these 6 SNPs revealing a protective effect against CIN; and a tendency of susceptibility to CIN appeared in H2 (similar to CCR5-HHE), H3 (similar to CCR5-HHF) and H4 (similar to CCR5-HHA), although these haplotypes did not show statistical association with CIN. The haplotype analysis results coincided with the results of our allelic and genotypic analysis and indicated that the *CCR5* promoter haplotype was associated with cervical lesions development. However the correlation between haplotype of 6 SNPs in this study and expression of CCR5 need to test and verify.

In 2016, Che et al indicated that CCR5 mRNA and protein expression levels were extremely higher in cervical cancer tissue than in matched normal control tissue, and downregulation of CCR5 inhibited cervical cancer growth and invasion [15]. Sales et al also presented similar results that the level of CCR5 was high in cervical cancer tissue and the expression of CCR5 could be regulated by inflammatory pathways [16]. However, no previous reports on the CCR5 expression level in CIN tissues were identified. So we performed the transcription factor binding site prediction in the *CCR5* promoter and try to find the possible mechanism of association of SNPs to development of cervical lesions. The computer algorithms suggested that there are many binding sites of distinct nuclear and transcription factors in the *CCR5* promoter, and the 6 SNPs studied were located in the binding region of specific transcription factors (Fig. 1). Nucleotide substitutions in the *cis-*regulatory region of a gene may cause a loss of binding of some nuclear proteins, or binding of some novel nuclear factors, or alteration in nuclear factor binding affinity. A previous study found that SNPs in human *CCR5 cis*-regulatory sequences were associated with alteration in DNA/nuclear factor binding. Mummidi et al found both G and T in − 2554 site (rs2734648) could bind to four nuclear factor complexes (NF4-NF7), but the affinities for NF5-NF4 complexes bound to the -2554 T were significantly greater than the binding affinities to -2554G [27]. Bream et al [33] also found that -2554 T bound p65, c-Rel, and p50 with greater affinity than -2554G. We predicted that rs2734648-T was located in the binding region of FOXP3, TFII-I, C/EBP-beta, NF-AT1 and PR–B. Therefore, the transcriptional status of rs2734648-G, especially rs2734648-GG, might be different. So we infer that rs2734648-GG is significantly associated with the susceptibility of CIN by affecting the transcription factors binding. Mummidi et al also found the wild-type C allele in − 1835 site (rs1800024) specifically bound to two novel nuclear factors NF2 and NF3, but the mutant-T allele lost the ability to bind nuclear factor NF3 [27]. The binding region of transcription factor GR-α contains two polymorphic loci rs1799988 (− 2135 T > C) and rs41469351 (−2132C > T). These two polymorphisms are located so closely, only at a 2-base-pair interval. For there is no polymorphism in rs41469351, so nucleotide substitution in rs1799988 might affect the transcription factor GR-α binding. In addition, Mummidi et al [27] found that CCR5 haplotypes conferred the haplotype-specific differences in the promoter transcriptional efficiency, of which the haplotype HHA showed the least promoter activity, whereas the transcriptional activity of HHF haplotype was the highest. That is to say the haplotype H1, similar to HHC, have a medium promoter activity. And haplotype H1 showed a significant resistance to CIN. So we infer that nucleotide substitutions such as rs2734648-GG, rs1799987-AA, rs1799988-CC and rs1800023 could associated with susceptibility to CIN by alternate nuclear factors binding,, then subsequently affect the efficiency of *CCR5* translation and *CCR5* expression level on cell surface and, finally, affect the development of cervical lesions. By the way, our results revealed that the susceptibility of SNPs in CCR5 promoter arising more in recessive inheritance model, such as rs2734648, rs1799987, rs1799988 and rs1800023. That is to say individuals carried two mutations in both alleles raise the susceptibility to CIN. This can be understood as the efficiency of nuclear factors binding, CCR5 translation and expression could be affected more when two mutations in both alleles of SNP.

In this study, we found the frequencies of the minor alleles of rs2734648 was significantly higher in the CIN group than that in the control group and the frequencies of rs2734648-GG, rs1799987-AA, rs1799988-CC and rs1800023-AA were distinctly increase the susceptibility of CIN. However, rare associations of susceptible to CC or from CIN progressing to CC were found. It is known that persisting high-risk HPV infection, which usually related to the lack of HPV-specific T-cell immunity and some immune-response pathway [11, 13], is associated with the development of vassal cell layer infection and hyperplastic lesions called cervical intraepithelial neoplasia (CIN). CIN lesions may regress, persist or progress to invasive cervical carcinogenesis [14]. Carcinoma development depends on different strategies to escape recognition by immune cells. So we think the mechanisms of normal cervical tissue progressing to CIN and CIN progressing to CC might be different, and the host immunogenetic factors played in development and progression of cervical cancer need to further study.

## Conclusion

In conclusion, we demonstrated that SNPs in the *CCR5* promoter are significantly associated with development of cervical intraepithelial neoplasia. These polymorphisms could potentially influence not only the CCR5 gene expression but also the CCR5 mRNA stability and the translation efficiency, which subsequently influence the density of CCR5 on cell-surface and, thus, have an impact on virus infection or disease pathogenesis. However, there might be some other mechanism of cervical cancer development, because our results indicated that polymorphisms in the *CCR5* promoter are not associated with progression from CIN to CC. Thus, sample size expansion for association study and the functional study on correlation of CCR5 polymorphisms with cervical intraepithelial neoplasia development, and finding other immunogenetic factors associated with cervical carcinoma will be performed in the future.

## Data Availability

All data generated or analyzed during this study are available to any scientist wishing to use them for non-commercial purposes from the corresponding author on reasonable request. But those clinical data are not available for authors have an ethical and legal responsibility to respect participant’s rights to privacy and to protect their identity.

## Abbreviations

AR: Androgen receptor
C/EBP: CCAAT/enhancer-binding protein
CC: Cervical cancer
CCR5: C-C motif chemokine receptor type 5
CIN: Cervical intraepithelial neoplasia
CTF: CCAAT box-binding transcription factor
CTF: Site-specific DNA-binding proteins or CAAT box transcription factor
E2F-1: Adenovirus E2 gene promoter region binding factor 1
EBF: Early B-cell factor
ENKTF-1: Enkephalin transcription factor-1
FOXP3: Forkhead box P3
GR-α: Glucocorticoid receptor
HIV: Human immunodeficiency
HPV: Human papillomaviruses
HWE: Hardy-Weinberg equilibrium
LD: Linkage disequilibrium
MCP: Monocyte chemotactic protein
MIP: Macrophage inflammatory protein
NF: Nuclear factor
OR: Odds ratio
PR: Progesterone receptor
RANTES: Regulated on activation, normal T cell expressed and secreted
SNP: Single-nucleotide polymorphism
SRF: Serum response factor
STAT4: Signal transducers and activators of transcription
TF: Transcription factor
XBP-1: X box binding protein 1
